# Assessing the outcomes of implantable cardioverter defibrillator treatment in a real world setting: results from hospital record data

**DOI:** 10.1186/1472-6963-13-100

**Published:** 2013-03-15

**Authors:** Simone Ghislandi, Aleksandra Torbica, Giuseppe Boriani

**Affiliations:** 1 Department for Policy Analysis and Public Management, Università Commerciale Luigi Bocconi, Milan, Italy; 2 Centre for Research on Health and Social Care Management (CERGAS), Università Commerciale Luigi Bocconi, Milan, Italy; 3 European Health Technology Institute for Socio Economic Research, Brussels, Belgium; 4 Institute of Cardiology, University of Bologna, Bologna, Italy

**Keywords:** Technology assessment, High cost technology, Matched paired analysis, Implantable cardioverter defibrillators

## Abstract

**Background:**

A plethora of clinical studies have assessed the benefits of implantable cardioverter defibrillators (ICDs) and supported their use in clinical practice. However, evidence on the safety and efficacy of ICDs appears insufficient to support expansion of their use in clinical practice, and more information on their impact in real life settings is warranted. This paper aims to investigate the impact of ICDs using a large administrative dataset reflecting actual clinical practice.

**Methods:**

Data were obtained from the hospital discharge database of the Friuli Venezia Giulia region in Italy containing patient-level information on 169,488 cases. Data on mortality outside hospital were obtained from regional sources. Exact matching method was used to estimate the outcomes associated with ICDs: mortality, length of stay, re-hospitalization and regional expenditure. The method was applied in two steps. First, patients with ICDs were matched with those without using the following: age class (by 5 years), gender, year of admission, type of admission (day hospital vs. ordinary) and primary diagnosis. In the second step, matching included also Charlson Comorbidities Index. Exact matching average treatment effect on the treated (ATT) was used as a main measure of impact.

**Results:**

Compared with matched controls, treatment with ICDs was associated with lower mortality (absolute risk reduction 10.6% at 1 year and 8.3% at 2 and 8.4% at 3 years, *p* < 0.001 and hazard ratio 0.80, *p* < 0.001), greater regional expenditure at index hospitalization (ATT: €9459.64, *p* < 0.001) and during follow up (ATT: €1707.29, *p* < 0.001) and higher re-hospitalization rate (ATT: 0.53, *p* < 0.001). No significant difference was found for length of stay (9.07 vs. 8.86 days). The results were maintained after more restrictive matching was applied.

**Conclusions:**

Assessing the impact of innovative, expensive medical technologies on the basis of real world data is warranted, especially when there are barriers to implementation. Hospital administrative datasets can be of great value when a technology such as the ICD is implemented in a relatively small sample of patients, to allow use of exact matching techniques.

## Background

Despite improvements in prevention and treatment strategies, cardiovascular diseases continue to represent a leading public health problem worldwide [1-4]. A plethora of studies have assessed the benefits of new technologies used in cardiology and support their use in clinical practice. However, accumulated evidence on the safety and efficacy of an innovation is not sufficient to support its widespread application. Owing to limited availability of the resources required for the use of new innovations, medical decisions are increasingly influenced by economic considerations. This is particularly evident in cardiology, where innovations are associated with significantly higher upfront costs that often create barriers to their use [5].

The implantable cardioverter defibrillator (ICD), introduced for the prevention of sudden cardiac death, is one of the most challenging examples in this regard. The deployment of ICDs in standard clinical practice has brought marked clinical benefits to the population at risk of fatal arrhythmias and dramatically changed the management of patients at risk of sudden cardiac death. The impact of ICDs on survival has been thoroughly investigated in numerous randomized clinical studies and the role of ICDs in preventing sudden cardiac death has been established for both secondary prevention (in patients who have already suffered a major cardiac event such as ventricular arrest) and primary prevention (in patients who are at risk of sudden cardiac death but have not yet suffered cardiac arrest). The weight of evidence supporting the clinical efficacy of ICDs in selected patients led to their inclusion in consensus guidelines issued by professional societies in both Europe and the United States [6]. A recent review suggests that ICDs are efficacious in reducing all-cause mortality in the adult population, with an effect ranging from 20% (95% confidence interval (CI) 10–29%) in randomized clinical trials to 46% (95% CI 32–57%) in observational studies [7].

However, evidence suggests that clinical guidelines are not always fully adopted owing to the presence of barriers to the widespread use of ICDs [8,9]. Their high upfront cost has been discussed as the major limiting factor: 88% of clinicians report financial barriers as the major obstacle, while lack of expertise and lack of national guidelines are perceived as potential barriers by 61% and 51% of clinicians, respectively [9]. Other factors that have been mentioned include organizational, administrative and cultural issues [10].

The literature on ICDs highlights two issues requiring further exploration. The first concerns the use of “real world” (i.e. observational) data for the evaluation of medical technology. This issue is particularly relevant for ICDs, because the reality can differ significantly from what is suggested by consensus guidelines. Validation of the potential benefits initially observed in clinical studies with data collected in real world medical practice is thus of crucial importance. Differences between the results obtained in these two settings can be of great value for healthcare providers, policy makers and producers of innovative technologies [11]. The second issue is related to the time over which analyses are conducted. Because ICDs are preventive devices, the importance of taking a long-term perspective when evaluating their impact is obvious. According to a recent review, however, no clinical trial has followed-up patients beyond 5 years. In particular, among published randomized controlled trials (RCTs), time limits of 4.5 years in secondary prevention and 3.5 years in primary prevention have been used [7].

These two issues (the need for real world data and a long-term perspective) have led to the establishment of several national and international registries to monitor and validate ICD use in routine clinical practice [12]. Data collected in these registries have given rise to numerous publications showing that adoption rates differ significantly across countries, even within the European Union. More specifically, according to national registries, it has been reported that, in 2008, the number of implants per million inhabitants was 70–90 in Sweden, the United Kingdom and Spain, 100–130 in France, Norway and Slovakia, 130–160 in Austria, Belgium and Switzerland, 160–200 in The Netherlands and Czech Republic, 228 in Denmark, 262 in Germany and 309 in Italy [13]. These differences suggest that attitudes toward the implementation of ICD therapy, especially in the context of primary prevention [8], are not homogeneous, again raising doubts about the generalizability of results from clinical trials and adherence to international guidelines.

Although studies based on registry data provide valuable insights into major differences in implant rates across countries, they are rarely used to evaluate the impact of ICDs [10,12-14]. The main problem here is that these studies do not generally identify any “control group”, with the obvious consequence that it is usually not possible to assess the short- and long-term effectiveness of the ICDs. An alternative approach is needed to better exploit information obtained from observational datasets.

The purpose of this paper is to fill this gap in the empirical literature. More specifically, the present study contributes to the existing literature in two ways. First, from a policy point of view, it provides a first quantitative assessment of the impact of an innovative technology (the ICD) in a real life setting. As mentioned above, this type of evidence is of paramount importance for decision making when allocating scarce healthcare resources. Second, from a methodological point of view, our paper approaches the impact evaluation exercise by directly using information from hospital records, originally collected for administrative purposes, from a region of Italy. The data used here are routinely stored by health authorities in many other European countries. Hence, the methodology proposed can be extended to analyze the impact of medical technology such as ICDs in different policy contexts and health systems.

The paper is organized as follows. In the next section, we describe the databases used, followed by essential descriptive statistics to set the context for the data analysis. In the subsequent section, we present the results, while the discussion is dedicated to interpretation and study limitations. We draw some conclusions in the final section.

## Methods

### Data source

The data were obtained from the hospital discharge database of the Friuli Venezia Giulia (FVG) region of Italy (Scheda Dimissione Ospedaliera, Major Diagnostic Category 5 – Disease and Disorders of the Circulatory System). These type of data are routinely collected by all the hospitals in the region and include information on patients socio demographic characteristics and treatment received during hospitalization.

FVG is in the northeastern part of Italy and has approximately 1.2 million inhabitants. As in the rest of Italy, all citizens are covered by taxed-based public health insurance. Since 1995, hospital care services delivered by public or private accredited hospitals in Italy are reimbursed on “per case” basis, classified according to Diagnosis Related Groups (DRGs). Each group is assigned a specific “value” (tariff) reflecting the intensity of resource consumption needed to treat patients assigned to that group. Costing for the purpose of tariff setting is mainly performed at regional level. Given that cost assessment represents essential part for defining value of DRG tariff, the latter is frequently used as the “proxy” of hospital costs of specific patient group.

We observed all patient records for the period 1997–2007 from all service providers in the region (24 hospitals). A total of 334,764 observations (admissions) and 169,488 patients were included in the analysis. For each observation, data included information on hospital diagnosis and procedures (International Classification of Diseases, 9th revision (ICD9), Clinical Modification codes), inpatient mortality, hospitalization and discharge dates, DRG tariffs and demographic variables (municipality of patient residence, age and sex). In addition, we merged this database with another regional administrative data set recording outpatient mortality from 1997 to 2009. This was particularly important in our context because, given the preventive nature of ICD treatment, inpatient mortality alone would have been a poor indicator of effectiveness.

As in other retrospective studies designed to assess current clinical practice [10], the current regulations of ethics committees in Italy require only standard written informed consent at the time of device implantation (obtained from all patients in line with national regulations) and anonymous publication of scientific data. Our retrospective observational study, conceived and performed in accordance with the principles of the most recent revision of the Declaration of Helsinki, fulfilled these requirements.

**Figure 1:**
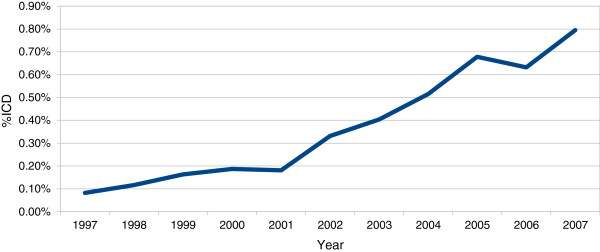
Diffusion of ICDs in Friuli Venezia Region 1997-2007 (% of patients with ICDs over total number of hospitalizations in MDC 5).

The average age at admission of all patients hospitalized in cardiology departments in FVG during the 11 years was approximately 70 years. Females represented 48.77% of the sample. On average, each patient stayed in hospital for approximately 9 days and was re-hospitalized 1.4 times. Overall in-hospital mortality was 5.5%. The total hospital expenditure per patient was €13,358 during the observation period, and the average DRG tariff for a single admission was €3395. Crucially, over the 10-year observation period, only 1213 ICDs were implanted (0.36% of the observed hospitalizations). Methodologically, this characteristic of the data is important, because it shows that ICD treatment is numerically a rare event and should be analyzed with the proper statistical tools. It is interesting to note, however, that the number of hospitalizations for ICD treatment increased steadily throughout the observation period, though it remained relatively low reaching maximum 0.80% in 2007 (Figure 1).

### Data analysis

To evaluate the impact of ICDs, we identified the following outcome variables: mortality, length of stay (LOS), re-hospitalization rate and regional expenditure. Mortality obviously represents an important final end point that directly reflects the effectiveness of ICD treatment. The other three quantities might be considered proxies of resource consumption associated with ICD use. From empirical evidence in the literature, we should expect mortality to be reduced when ICDs are implanted. Simultaneously, we should expect expenditure to be increased for ICD patients. We did not have any particular *a priori* expectations regarding how long patients treated with an ICD might stay in hospital (LOS) or how many times they might have to be re-hospitalized.

ICD implantation is a rare event. Evidently, direct comparison between ICD patients and the rest of the sample would not be informative because the ICD patients would be compared with a very large and heterogeneous control group comprising patients hospitalized for a wide variety of different pathologies and health problems. This could significantly bias the results. For example, treatment with an ICD could be associated with higher than average mortality simply because ICDs are used in people at high risk of future heart failure.

The general approach taken in this paper was to use exact matching methodology to select a subsample of homogeneous records. The variables used for matching were: age class (i.e. age stratified in classes of 5 years), gender, year of admission, type of admission (day hospital vs. ordinary), primary diagnosis and comorbidities. Exact matching is a relatively straightforward technique and is particularly suited to situations in which the treatment is a rare event [15,16].

In our case, for each patient who received an ICD (treatment group), we selected a group of patients with the exactly same characteristics (control group) and compared their outcomes with simple mean difference analyses. More specifically, in our analysis we evaluated what is known as the average treatment effect on the treated (ATT), which, in this case, measures the impact of the ICD on the people that received one. The method adopts the following approach: once the subsample of patients with the same characteristics had been selected, the impact of the ICD on a certain outcome was measured by comparing the outcome for each single ICD patient (case) with the average outcome of all of the matched patients without the ICD (control). As specified above, matching was done on exact values. This means that each single case was compared with a control with exactly the same characteristics (in terms of covariates), but with an “artificial” outcome obtained by averaging all of the possible matched controls. In this method standard errors and the *t*-test are not straightforward to apply and need adjustments. For this reason, we used the *nnmatch* STATA command, which calculates the correct variance and CIs [17].

The most important variables for matching are the primary diagnosis and comorbidities, which in our dataset was codified according to the ICD9-CM. Each hospital admission was associated with up to six of these codes. Because there are many possible combinations of six variables, we followed a two-step matching procedure. First, patients were selected according to their primary diagnosis; that is, we selected a control group of patients who had exactly the same primary diagnoses as the corresponding ICD patients (subsample 1). In the second step, we accounted for comorbidity by calculating the Charlson comorbidity index (CCI) for the ICD9 classification. This index provides a numerical stratification of the severity of the comorbidities associated with the diagnostic codes. In our case, the CCI was applied to the diagnoses not used in the first step (diagnosis two to six). Once the CCI index for each observation was obtained, further matching was applied (subsample 2).

Since we observed hospitalizations to the end of 2007 and outpatient mortality to the end of 2009, follow-up ranged from a minimum of 2 years to a maximum of 10 years. However, since the year of hospitalization is included in the matching process the follow-up period, by definition, could not differ significantly between the treatment and the control group. In our analysis we observe mortality at different points in time following hospitalization (at 1, 2 and 3 years).

It is important to distinguish between the subsample used for analysis and the sample used matching. Because matching builds a 1:1 counterfactual, the case and control groups are by definition numerically equal and perfectly balanced in their covariates. However, the selected subsample does not need to have these characteristics since the number of controls for each case may vary.

Regarding the assessment of mortality, since the day of death cannot be “averaged” in the control group, exact matching cannot provide an analysis of the hazard ratios related to ICD use. Consequently, it is reasonable to assess the robustness of our results from exact matching by applying a regression-based parametric approach to the selected subsamples. We applied a Weibull regression analysis on the selected subsamples, using the diagnostic variables as controls.

Finally, it should be noted that ICDs were available from only eight of the 24 hospitals in FVG. One might have been tempted to use hospital as a further matching variable. However, if an ICD is not implanted in a center where this treatment is available, it is likely that the decision depended on factors known to the doctors but unobserved by the analyst (see the last section for a discussion of this issue). Hence, one possible selection strategy is to pick the control group only from those hospitals were ICDs were unavailable. This reduces the risk of no-ICD being dependent on unobserved factors, and is the approach we took in the following analysis, though it should be noted that the results change little if hospital is used as a matching variable.

## Results

Table 1 reports main descriptive statistics on the two subsamples before and after matching. Subsamples 1 and 2 were obtained by considering different specifications of the diagnosis variable, as described in the previous section. When only the primary diagnosis is considered (subsample 1), 1050 patients, from a total of 13,029, received an ICD. Matching for CCI halved the size of the control group (5962 vs. 11976), whereas the treatment group was reduced to 905 units.

**Table 1:** Before and after matching sample characteristics

	**Sub-sample 1**			**Sub-sample 2**		
	**Before matching**		**After-matching**	**Before matching**		**After-matching**
	**Mean case (sd)**	**Mean control (sd)**	**Standardized difference**	**Mean case (sd)**	**Mean control (sd)**	**Standardized difference**
***Covariates***						
Age	65.90 (12.02)	70.40 (10.09)	−0.01	66.30 (10.58)	70.12 (9.83)	−0.01
Gender (Female)	0.15 (0.35)	0.16 (90.38)	0	0.14 (0.35)	0.15 (0.35)	0
Charlston Index = 0				0.53 (0.49)	0.54 (0.50)	0
Charlston Index = 1				0.24 (0.43)	0.30 (0.46)	0
Charlston Index = 2				0.14 (0.35)	0.21 (0.41)	0
***Outcomes***						
LOS	9.15 (11.9)	9.05 (9.72)	0.152	8.86 (11.24)	9.07 (9.40)	0.154
Re-hospitalization rate	2.11 (3.51)	1.54 (2.52)	0.202	2.14(3.60)	1.57(2.42)	0.227
Mortality	0.29 (0.45)	0.47 (0.50)	−0.109	0.30 (0.46)	0.46(0.50)	−0.067
Expenditure for index hospitalisation (€)	15020.92 (5780.23)	4259.72 (5780.95)	1.44	14815.56 (7343.40)	4307.13(6443.55)	1.347
Total hospital expenditure during follow up (€)	24944.31 (19558.93)	10881.34 (13230.06)	0.156	24829.44 (19863.8)	11269.01 (14044.27)	0.618
Patients	1050	11976		905	5962	

Legend: **Sub-sample 1**: ICD and non ICD patients with the same primary diagnosis (+ other controls); **Sub-sample 2**: ICD and non ICD patients with the same primary diagnosis and Charlson index (+ other controls; **Case:** Patients with ICD; **Controls:** Patients without ICD; **Standardized difference =** After-matching standardized differences between case and control groups; **LOS** = Length of Stay in day.

Considering after-matching sample characteristics, average age changed little, whereas the proportion of male patients was approximately 80%. This is because, in the original dataset, only approximately 16% of the patients who were given an ICD were female. Thus, proportions were maintained in the matched sample also with regard to gender. The most common primary diagnosis among patients with ICDs was primary cardiomyopathy (41%), followed by paroxysmal ventricular tachycardia (23%). A secondary diagnosis was registered in 60% of the ICD patients, with congestive heart failure in 30% of cases.

Table 1 also shows after-matching standardized differences between the treatment and control groups. Note that, because we used exact matching, a balance among the covariates was by definition always achieved. It was not null in the “age” covariate because we used 5-year age classes instead of actual age for matching. However, there was clearly no balancing problem. The after-matching standardized differences for the outcomes (LOS, re-hospitalization rate, mortality and expenditure) in Table 1 give an initial idea of the size and the sign of the impact that we measure more precisely below.

Results for series of outcomes are reported in Table 2. Mortality at 1, 2 and 3 years was significantly lower in patients with ICDs. Clearly, the impact of ICDs was accentuated in the first year after implantation; the benefit from ICD treatment in terms of mortality avoided remained rather stable between the second and third years. Note that the average treatment effect on mortality is a measure of the absolute risk reduction with an ICD. Given that the mortality in the matched control group was approximately 18% at 1 year, 27% at 2 years and 45% at 3 years, the data in Table 1 imply relative risk reductions of 0.59, 0.30 and 0.16, respectively. We also determined the impact of ICDs on the number of life years gained (LYG). This is calculated as minus the case–control difference in the life years lost due to death, that is, the difference between the age-sex specific life expectancy in Italy in 2007 and age at death. This measure assumes that patients surviving the observation period will, on average, live as long as the rest of the population. Our results show that an ICD is associated with significantly longer life expectancy (ranging from 0.762 years in matching 1 to 1.190 in matching 2).

**Table 2:** **Average treatment effect on patients with ICDs (adjusted *t*-test)**

	**Average treatment effect on ICD patients**	**95% confidence interval**	
		**Lower**	**Upper**
***Matching 1***			
Mortality 1 year	−0.106	−0.131	−0.082
Mortality 2 years	−0.083	−0.114	−0.053
Mortality 3 years	−0.084	−0.120	−0.049
Life years gained	1.190	0.657	1.723
LOS	0.112	−0.853	1.078
Re-hospitalization	0.533	0.325	0.742
Expenditure for index hospitalization (€)	9459.64	8793.73	10,125.54
Total hospital expenditure during follow up (€)	1707.29	539.25	2875.33
***Matching 2***			
Mortality 1 year	−0.088	−0.114	−0.062
Mortality 2 years	−0.065	−0.097	−0.032
Mortality 3 years	−0.060	−0.099	−0.021
Life years gained	0.762	0.202	1.323
LOS	0.089	−0.648	1.112
Re-hospitalization	0.591	0.360	0.818
Expenditure for index hospitalization (€)	9103.54	8543.86	9981.10
Total hospital expenditure during follow up (€)	1747.94	379.70	2997.16

Legend: **Matching 1:** exact matching on subsample 1**; Matching 2**: exact matching on subsample 2; **Average treatment effect**: average treatment effect on patients with ICDs (mean difference between treatment and matched controls, adjusted for number of matches); **Mortality 3 years**: patients hospitali*z*ed in 2007 excluded.

No significant difference between treatment and control groups was found for LOS. However, re-hospitalization rates were higher for ICD patients in both matching exercises (Table 2).

Similar results were obtained for regional expenditure. In our analysis, regional expenditure is reflected in the value of DRG tariff paid to the hospital for each patient included in the subsample. Differences in expenditure between the treatment and control groups at index hospitalization were almost constant at €10,000, which reflects the difference in DRG tariff when the ICD is initially implanted. However, this difference drops significantly during the follow up. In both matching steps, the expenditure during the follow up for ICD patient is approximately €1700 higher than the control group, with no significant differences between different matching approaches. This pattern reflects higher re-hospitalizations in patients with ICD implanted, as mentioned above.

The same subsamples can be used to perform a robustness check for the non-parametric matching described above. Table 3 depicts a Weibull hazard model of the two subsamples. Remember that, though the subsamples were selected to have the same characteristics as the patients who received ICDs, the distribution of these characteristics might vary between the treatment and control groups. Hence, it is fundamental to control for the same variables when performing a parametric analysis. This is why we continued to use the CCI and primary diagnosis as covariates. Also note that the Weibull *p* is always significant and smaller than 1, indicating that the risk of death declines over time. Results show a hazard rate of 0.80 and 0.85 in subsample 1 and 2, respectively.

**Table 3:** Hazard ratio for subsamples (Weibull hazard model)

	**Subsample 1**			**Subsample 2**		
	**Hazard**	**95% CI**		**Hazard**	**95% CI**	
ICD	0.807	0.710	0.916	0.848	0.747	0.985
Age	1.053	1.049	1.057	1.057	1.049	1.051
Sex	0.705	0.651	0.764	0.681	0.651	0.601
Charlson comorbidity index	1.416	1.369	1.465	1.341	1.279	1.406
Weibull *p*	0.81	0.791	0.830	0.807	0.780	0.835
Likelihood Ratio (LR) (*p*-value)	3436.31 (< 0.0000)			1604.94 (< 0.0000)		

All models included 48 dummies for primary diagnosis (coefficients not shown here); ICD: whether patient received an ICD (1) or not (0).

## Discussion

It was argued that real word data are more informative and useful for decision makers than results from controlled trial settings when evaluating medical technologies. This is particularly relevant in situations in which clinical practice may significantly differ from clinical guidelines owing to the presence of barriers to the full implementation of technologies in clinical practice [14]. This effect has been observed in the introduction of ICDs for the prevention of sudden cardiac death. To our knowledge, this is the first empirical study that has attempted to determine the impact of ICDs using an administrative data set from a real world setting in Europe. Administrative databases are recognized as valid sources of data for identifying the outcomes of rare events and for assessing the economic impact of various interventions [11,18]. We believe that this analysis contributes to the existing literature in two ways. First, it provides additional evidence regarding the impact of ICDs and estimates the magnitude of this impact across four different dimensions. Second, and even more importantly, it represents a novel study design in which an exact matching method was used.

In this study, ICD treatment was associated with significantly lower mortality, slightly higher re-hospitalization rate and significantly higher regional expenditure. In our sample, mortality at 1 year was reduced by between 9% and 10%, with a relative risk reduction of 0.59. The hazard ratios for the two subsamples were 0.80 and 0.85, respectively. These findings are in line with those reported from RCTs and collected in recent reviews, though direct comparisons should be made with caution, considering the different study designs [18]. A more significant difference is observed in comparison with the results obtained from a meta-analysis of other observational studies in which ICDs reduced all-cause mortality by 46% (CI, 32% to 57%) [7]. This greater difference can be partially explained by the high heterogeneity of the studies selected for this review. Indeed, among 11 observational studies with a contemporaneous control group, only two can be compared with our present study in terms of sample size and the method used to determine the benefits of ICDs [19,20]. In line with our findings, the relative risk reduction in all-cause mortality in these two studies was 0.67 (CI 0.63–072 ) and 0.71 (CI 0.51–0.97), respectively.

Regarding re-hospitalization rate, the difference between the two groups was positive across all samples. This result could have been expected, because ICD patients are more likely to return hospital for monitoring of their device, though such visits do not necessarily become hospitalization events.

The study of Groeneveld et al. [19] analyzed Medicare patients given an ICD prophylactically and found increases in both survival and expenditure compared with propensity score-matched elderly patients who did not receive an ICD. This analysis differs from ours in terms of the method, the patients’ age (mean 76 years) and the context. The purchase price of an ICD is higher in the United States than in Europe, and specifically in Italy [20,21], and therefore American data cannot be directly related to the setting of our health care system. However, it is noteworthy that these two studies led to similar conclusions with regard to ICD costs and benefits. More recently, another report derived from Medicare data showed how geographical areas in which the prophylactic use of ICDs increased over time showed greater improvements in survival, stressing the need for programs designed to increase the evidence-based use of ICDs [22].

In our study, regional expenditure was significantly higher for ICD patients both at the index hospitalization and during follow up. Both differences were driven mainly by the fixed difference between the two predominant DRG tariffs in the two groups. These estimates provide an insight into the economic impact of ICDs. In fact, given that cost assessment represents essential part for defining value of DRG tariff, the latter is frequently used as the “proxy” of hospital costs of specific patient group. However, it has been argued that costing is not the only ingredient in determining DRG tariffs and that DRG tariffs may not fully reflect the actual resource consumption associated with patient’s management [23]. To estimate actual cost per patient, we would need further data on the resources used and their unit costs, which were not available in the present study.

The finding of similar LOSs and costs during follow-up in patients who did and did not receive an ICD is of interest, because in ICD patients prevention of sudden cardiac death by termination of ventricular tachyarrhythmia has been found to be associated with a subsequent increased risk of heart failure, with a potential risk of increased hospitalization for heart failure [24]. In RCTs, the proportion of non-sudden deaths (including heart failure) showed a relative increase in patients given a prophylactic ICD compared with controls, though the absolute number of heart failure deaths was not increased [25].

A criticism of our study is related to the interpretation of the results in terms of the causal relationship between ICDs and mortality. Clinicians treat patients with ICDs according to unobservable factors, some of which might be correlated with pre-treatment mortality. Thus, because we do not know everything about clinicians’ decisions, our treatment and control groups might differ in some unobservable characteristic. This possibility cannot be excluded and is an important issue when interpreting the evidence. However, two features of our study must be highlighted in this regard.

First, though selection based on unobservables is an intrinsic problem of non-experimental evidence, the relevance of our results does not depend on the correct identification of a causality relationship (which can be achieved only through proper clinical trial designs). From a health policy perspective, our results should be interpreted as important evidence that the real world use of a new medical technology, the ICD, is in line with the clinical evidence and that the health system is not significantly distorting the application of this effective but expensive preventive technique. It is noteworthy that a systematic review and meta-analysis published by Ezekowitz et al. found that mortality over time was similar between ICD patients enrolled in RCTs and those in observational studies (both prospective and retrospective) [7].

Second, if the external validity of an RCT must be verified, observational data represent the best description of the physicians’ behavior and patients’ outcomes. A certain degree of bias, excluded by the design of RCTs, is thus unavoidable in this context. Alternatively, some might prefer to use a more structured approach in which the treatment is assumed to be assigned according to pre-specified criteria, generally coming from a theoretical model (e.g. see Heckman [27]). However, these methodologies were primarily developed for cases in which the analyst has substantial information about each individual (e.g. obtained from a survey) and can use extra matching variables to predict selection for treatment. Here, we know only what we could observe from administrative datasets and used all the available information for matching. Hence, we see no further advantage in introducing heavy discretionary assumptions about the selection process in this context.

## Conclusions

Although we acknowledge its limitations, we believe that the present paper contributes to the existing literature by illustrating the potential use of administrative datasets in evaluating the benefits of the implementation of new technologies in the real world. There is huge potential in using these data that is often underestimated in the scientific literature. In the specific case of ICDs in the real world setting of a region of Italy, ICD therapy was associated with a positive impact on mortality in comparison with patients matched for age and administrative and clinical variables. The survival benefit was associated with greater expenditures for the index hospitalization, but costs were similar during follow-up.

## Abbreviations

LYG: Life years gained; ATT: Average treatment effect on the treated; ICD: Implantable cardioverter defibrillators; CCI: Charlson comorbidity index; FVG: Friuli Venezia Giulia.

## Competing interests

All three authors declare that they have no competing interests.

## Pre-publication history

The pre-publication history for this paper can be accessed here:

http://www.biomedcentral.com/1472-6963/13/100/prepub
